# Revisional Laparoscopic One Anastomosis Gastric Bypass for Weight Loss Failure after Restrictive Procedures

**DOI:** 10.1007/s11695-026-08821-z

**Published:** 2026-07-06

**Authors:** Hossam S Abdelrahim, Ahmed Elnabil-Mortada, Wadie Boshra Gerges, Haitham M. Elmaleh

**Affiliations:** Ain Shams University Faculty of Medicine, Cairo, Egypt

**Keywords:** Revisional one anastomosis gastric bypass, Restrictive bariatric surgery, Suboptimal initial response, Late postoperative clinical deterioration.

## Abstract

**Introduction:**

Patients undergoing primary restrictive bariatric metabolic surgery procedures (BMS) are at risk of having suboptimal initial response (SIR) and late postoperative clinical deterioration (LPCD), which can be an indication for conversion. Roux-en-Y gastric bypass (RYGB) is currently the most common conversion procedure. Still, one anastomosis gastric bypass (OAGB) has emerged as a viable alternative conversion procedure to RYGB, due to its relative technical simplicity and easy reversibility. We aim to assess the safety and effectiveness of laparoscopic OAGB as a conversion procedure for managing SIR or LPCD after primary restrictive BMS procedures.

**Methods:**

A retrospective observational study was conducted at our university hospital between July 2017 and December 2024. It included patients with failed primary restrictive bariatric procedures. All patients underwent conversion to OAGB and were followed for a minimum of 5 years.

**Results:**

115 patients completed the 5-year follow-up. The early postoperative complication rate was 7.6%, and the reoperation rate was 1.6%. After 5 years, late postoperative complications occurred in 11.3% of patients. The mean body mass index loss (BMIL%) was 28.59% and 17.4% of patients had SIR during the study period. Significant improvement in obesity-associated medical problems was observed after 5 years, with remission rates of 70.8% for type 2 diabetes mellitus, 82.4% for dyslipidemia, 64.1% for sleep apnea, and 61.3% for hypertension.

**Conclusion:**

OAGB is a safe and effective conversion procedure for the management of SIR and LPCD after restrictive procedures, achieving favorable mid-term outcomes.

## Key Points

• 115 patients of failed restrictive procedure underwent cOAGB with 5-year follow-up.

• cOAGB is a safe and effective conversion procedure.

• cOAGB has good metabolic outcomes.

## Introduction

Bariatric metabolic surgery (BMS) is currently the most effective long-term treatment for obesity and its associated medical problems. Bariatric procedures are traditionally classified as restrictive (procedures which lead to a physical reduction in the size of the upper gastrointestinal tract), malabsorptive (procedures which bypass a proportion of the intestine with less physical restriction of food intake), or a combination of both [1].

Primary restrictive procedures, including vertical band gastroplasty (VBG), laparoscopic adjustable band (LAGB), greater curvature plication (GCP), and laparoscopic sleeve gastrectomy (LSG), are simple, safe, and effective. However, they carry a risk of suboptimal initial response (SIR) and late postoperative clinical deterioration (LPCD), as well as other complications, that may necessitate conversion [2]. Cumulative failure rates after primary restrictive procedures range from 30% to 60% at five years [3]. The conversion rate following BMS is reported to be between 10% and 25% [4].

RYGB is currently the most common conversion procedure [2]. OAGB has been described as a viable conversion alternative, due to its relative simplicity and easy reversibility [5]. Primary OAGB has shown comparable or superior effectiveness to RYGB for weight loss and remission of type 2 diabetes mellitus (T2D), while being associated with shorter operating time and slightly fewer postoperative complications. Several studies have demonstrated the efficacy of OAGB as a conversion procedure [6]; however, most included small cohorts or did not specifically address outcomes after a restrictive procedure, limiting firm conclusions. In our study, we aimed to evaluate the safety and effectiveness of OAGB as a conversion procedure for managing SIR or LPCD after primary restrictive BMS procedures.

## Materials and Methods

### Study Design

This retrospective observational study (retrospective analysis of prospectively collected data) was conducted between July 2017 and July 2024 at our university hospital. Informed written consent was obtained from all participants included in this study. The study was approved by the Ethical Committee of the General Surgery Department (our IRB, Approval number 0006379).

### Study Population

The study included 150 patients.

### Inclusion Criteria

- Patients with failed primary restrictive bariatric procedure (VBG, LAGB, LSG, GCP).
- Age 18–65 years.
- Body mass index (BMI) ≥ 35 kg/m^2^.

### Exclusion Criteria

- Patients with procedure-related complications (e.g., band erosion, postoperative leakage).
- Patients with any endocrine abnormalities (e.g., hypothyroidism and Cushing’s syndrome).
- Pregnant or lactating females.
- Patients with major psychiatric illness, alcohol, or substance abuse.
- Patients with severe gastro-esophageal reflux disease (GERD) (grade B and C according to Los Angeles classification).

### Definition of Failure

According to IFSO Consensus [7], we used the following definitions in our study:

- Suboptimal initial response (SIR): inadequate weight loss is demonstrated by total body weight loss (TBWL%) or BMIL% of less than 20%.
- Late postoperative clinical deterioration (LPCD) is demonstrated by recurrent weight gain of more than 30% of the initial surgical weight loss.
- Conversion surgery: Surgical or endoscopic procedures to convert a new type of metabolic/bariatric operation.

Although updated terminology (SIR/LPCD) was applied during manuscript preparation, the operational definitions and outcome measures remained consistent throughout the study period.

### Preoperative Workup

- Complete clinical assessment and anthropometric measures.
- Full Laboratory and other relevant investigations.
- Nutritional & psychological assessment.
- Optimization of any co-morbid conditions.
- 3D- CT volumetry with gastrograffin meal.
- Upper gastrointestinal endoscopy (UGI).

### Surgical Techniques

All operations were performed laparoscopically using a minimum of 5 ports. The operative approach varied depending on the patient’s previous bariatric procedure.

In prior LSG, the sleeve was dissected free up to the angle of His. A window was made in the lesser omentum just proximal to the antrum. Through this window, an Echelon Flex 60 ™ stapler with a green or black cartridge (Ethicon) was fired in a horizontal direction. Then, a vertical tube was formed through sequential firings of stapler loads along a 36 French calibration tube (bougie), upward to the angle of His.

In prior LAGB, the band was removed, and the fibrous ring around the stomach was excised. The upper greater curvature was freed up to the left crus, then the gastric pouch was created as described before.

In prior VBG, we tried to pass a 36 French bougie tube from the pouch outlet. If passed easily, the first staple fire was placed as usual and subsequent fires were done along the old staple line. If pouch outlet obstruction was found, mesh removal was attempted cautiously. In case of successful mesh removal confirmed by easy passage of the bougie, the pouch was fashioned as usual. If we failed to remove the mesh, the first transverse firing site (supposedly just above the mesh) was assessed. If this site allowed for creating a pouch of at least 15 cm in length, the operation was completed as usual. If creating a long enough pouch was not possible, we would opt to perform an RYGB instead, and the patient was excluded from the study.

In prior GCP, the plication suture line was disrupted, then a calibrated (36 F) pouch was created using green/black reloads, removing plicated gastric tissue and sutures.

After pouch creation, the total small bowel length (TSBL) was measured from the ligament of Treitz. The bypass was set to be about 25% of the TSBL (a minimum 200 cm biliopancreatic limb, and a minimum 300 cm common channel were ensured). An antecolic, antegastric, terminolateral gastrojejunostomy was performed using a 45 mm blue load (the full length of the load was fired, creating a 3–3.5 cm wide anastomosis).

### Postoperative Care

Clear fluid diet was started on day one. A gastrografin swallow was done on day 2 to exclude leak. Patients were discharged once they were tolerating oral fluids and ambulating. Outpatient clinic follow-up visits were scheduled on postoperative day 10 and 1, 3, 6, 12, 18, 24 months, then annually up to 5 years after the operation.

Primary outcomes (5-years endpoint) were: (1) Weight loss (BMI, BMI loss %, TBWL %). (2) Resolution/improvement of obesity-related comorbidities according to the following criteria: dyslipidemia (normal lipid profile), hypertension (systolic blood pressure < 130 mmHg & diastolic blood pressure < 85 mmHg on 3 successive readings over 2 visits, off antihypertensive medications), and T2D (Hb A1C < 6.5% and fasting blood sugar was < 126 mg/dl, with no postoperative diabetic medication).

The secondary outcome measures were late postoperative complications during the follow-up period, including: - GERD: according to The Montreal Definition, diagnosis was based on a combination of symptom presentation, endoscopy, pH & manometric studies [8, 9]. - Bile reflux: diagnosed by demonstrating esophagitis or gastritis associated with the presence of bile on endoscopy, together with “non-acid” reflux in pH-impedance test. - Anemia by Hb levels < 13 gm/dL in adult men, and < 12 gm/dL in adult nonpregnant women. - Micronutrient deficiency was diagnosed by laboratory detection of deficiency of any of the following: Calcium, iron, zinc, Vitamin B1, Vitamin B12, Vitamin D.

### Statistical Analysis

Data were collected and analyzed using SPSS software (Statistical Package for the Social Sciences, IBM Corp., Armonk, NY) for Windows, Version 21. Quantitative variables were described using means and standard deviations (SD), and qualitative variables were described using frequencies. Statistical tests included the chi-square (X2) test, Student’s t-test, and ANOVA. Multivariable analysis was conducted using a logistic regression model. Significance levels were set at *p* < 0.05 (S), highly significant (HS) with *P* < 0.01, and Non-significant (NS) with *P* ≥ 0.05.

## Results

### Patients’ Characteristics

**Fig. 1:**
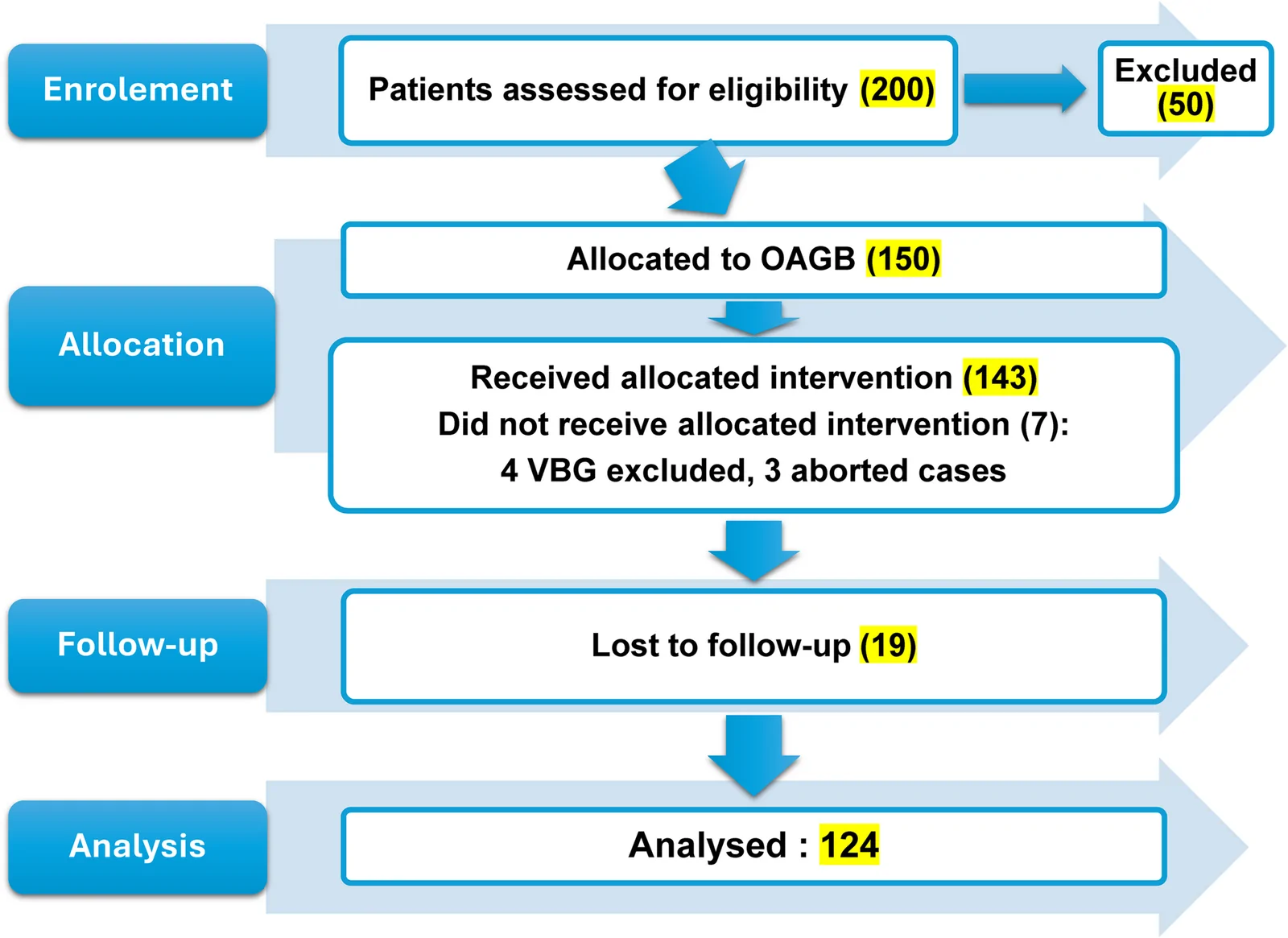
Study selection flow chart

200 patients with failed restrictive procedures were assessed for eligibility. 50 patients did not meet our eligibility criteria and were therefore excluded from the study. The remaining 150 patients were enrolled in the study. 4 VBG patients were excluded due to inability to convert to OAGB (they were converted to RYGB instead). The operation was aborted in 3 patients due to high airway pressure after insufflation. 124 patients were included in the first analysis of the study as shown in (Fig. 1). Table 1 shows the preoperative parameters among the study group.

**Fig. 2:**
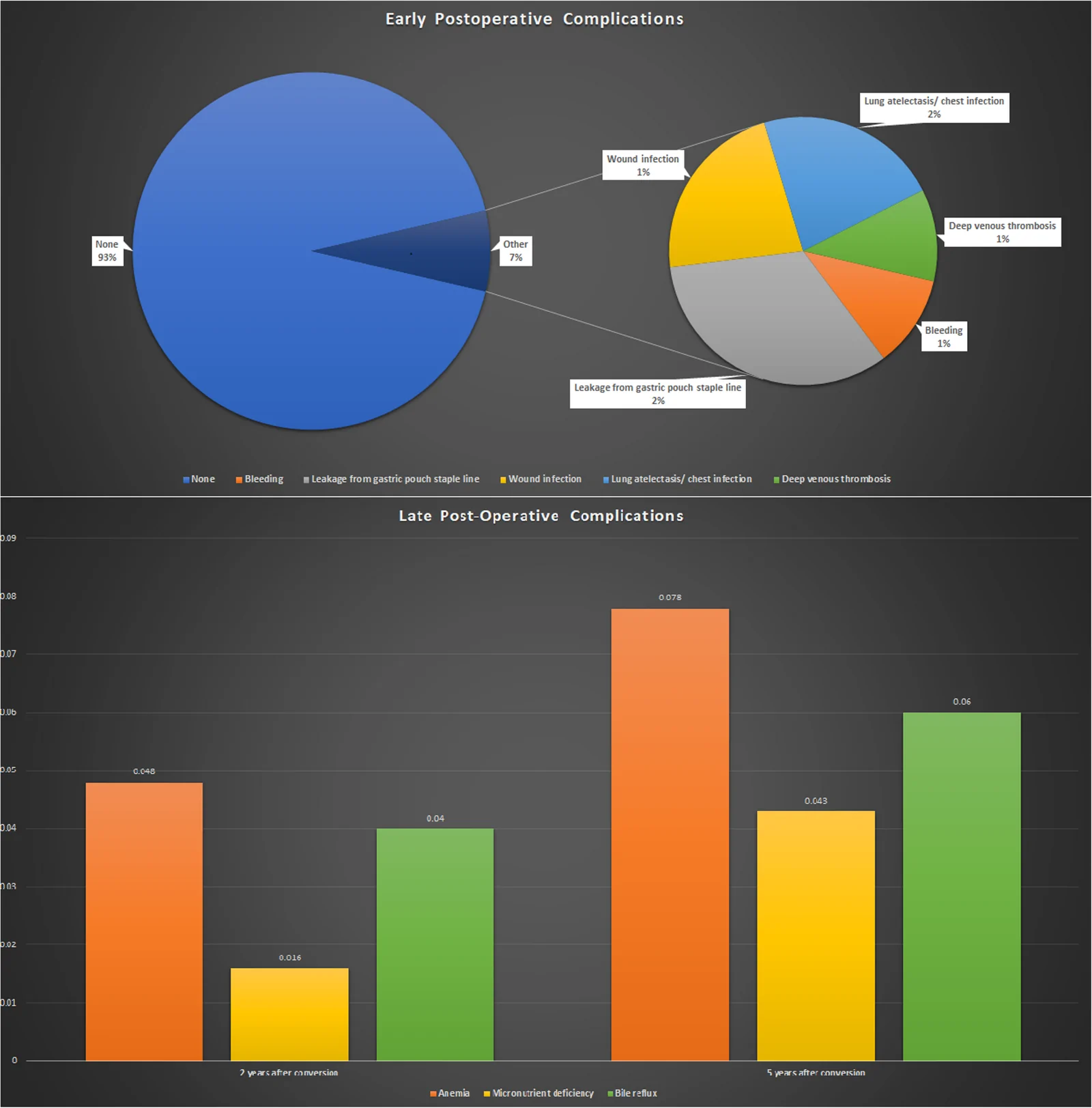
Early and late postoperative complications

**Fig. 3:**
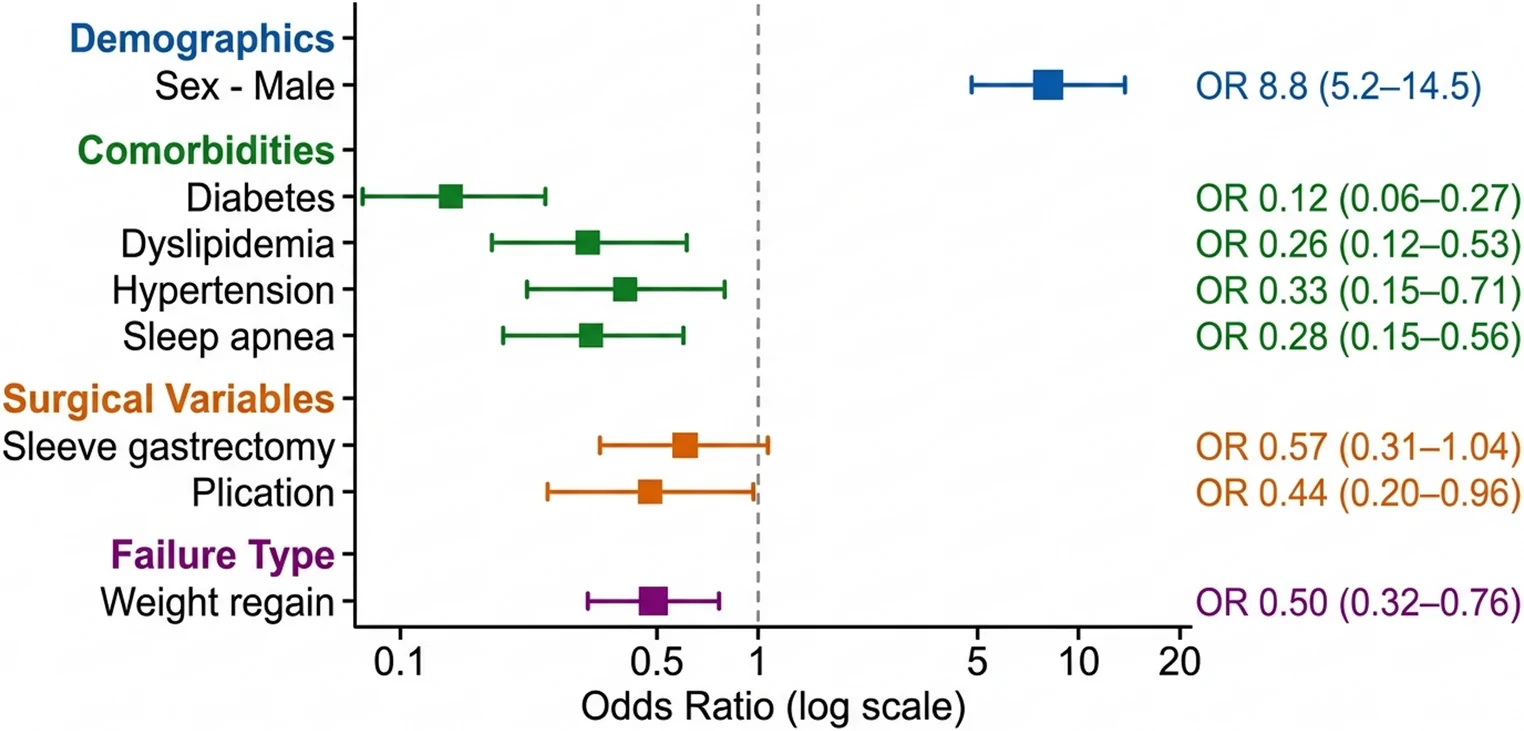
A Plot chart showing the results of multivariate logistic regression analysis to define the independent predictors of failure

**Table 1:** Preoperative parameters among the study group

Variable (124)	Value
Sex	
Female	72 (58.1%)
Male	52 (41.9%)
Mean age	42.4 ± 9.6 years (range 22–56)
Mean pre conversion weight	122.77 ± 18.25 Kg (range 96–183)
Mean pre conversion BMI	48.72 ± 13.47 kg /m^2^ (range 39.7–64.8 kg/m^2^)
Mean duration between primary operation and conversion	42.12 ± 15.81 months (range 23–87)
Type of primary surgery	
LSG	26 (21%)
VBG	56 (45.1%)
LAGB	31 (25%)
LGCP	11 (8.9%)
Indication for conversion	
SIR	71 (57.3%)
LPCD	53 (42.7%)
Associated comorbidities	
Hypertension	34 (27.4%)
DM	26 (21.0%)
Sleep apnea	68 (54.8%)
Osteoarthritis	73 (58.9%)
Dyslipidemia	37 (29.8%)

*DM *Diabetes mellitus, *BMI *Body mass index, *LSG *Laparoscopic sleeve gastrectomy, *VBG *Vertical band gastroplasty

*GCP* Greater curvature plication, *AGB* Adjustable gastric band, *SIR* Subclinical initial response, *LPCD* Late postoperative clinical deterioration

### Operative Data

All but 4 (3.2%) patients were completed laparoscopically. Conversion to open surgery was due to either extreme adhesions or significant intraoperative bleeding. Accidental iatrogenic enterotomies in 3 patients (2.4%) were identified and repaired during the operation. The mean operative time was 155.3 ± 20.6 min, and the mean intraoperative blood loss was 245 ± 55 ml.

### Postoperative Course, Morbidity & Mortality

The mean length of hospital stay was 5.2 ± 1.6 days. Early postoperative complications occurred in 9 patients (7.6%) as shown in (Fig. 2).

All early postoperative complications were successfully treated conservatively, except for one patient who had a gastrojejunal anastomosis leakage, which required conversion to RYGB, and another patient who had a combined wound infection with complete wound dehiscence, which required reoperation with closure of the wound using the anterior component separation technique. The overall reoperation rate was 1.6%.

9 patients were lost to follow-up during the 5-year follow-up, leaving 115 patients included in the final analysis.

Late postoperative complications were encountered in thirteen patients (11.3%) during follow-up as shown in (Fig. 2). All of them were successfully managed by nutritional and medical treatment. We had no cases of anastomotic stenosis/ulceration, internal hernia, or mortality throughout the study.

### Outcome Measures

#### Weight Loss

5 years post-conversion, the mean BMI decreased to 34.79 Kg/M^2^. SIR was detected in 20 patients (17.4%) during follow-up. Weight loss data are shown in Table 2.

**Table 2:** Body weight, BMI, BMIL%, EWL% and TBWL% data within the 5 years follow-up

Variable	Weight (Kg)		BMI (Kg/M^2^)		BMIL%		EWL%		TBWL%	
	Mean ± SD	Range	Mean ± SD	Range	Mean ± SD	Range	Mean ± SD	Range	Mean ± SD	Range
Pre - conversion	122.77 ± 28.25	96.00–183.00	48.72 ± 13.47	39.70–64.80	N/A	N/A	N/A	N/A	N/A	N/A
12 months	98.03 ± 23.48	76.53–143.63	36.73 ± 9.05	27.38–51.74	24.61 ± 5.51	20.15–31.03	49.74 ± 12.36	23.56–93.97	20.2 ± 19.1	16.9–37.7
24 months	90.31 ± 20.84	71.93–129.16	30.03 ± 7.79	24.17–43.7	38.36 ± 3.27	32.65–39.19	60.56 ± 14.15	43.16–113.34	26.4 ± 17.0	5.2–41.4
36 months	88.67 ± 15.41	69.12–127.57	29.01 ± 4.89	19.25–42.2	40.46 ± 8.23	34.88–51.52	63.11 ± 13.76	45.22–121.15	27.8 ± 12.6	3.9–43.7
60 months	102.6 ± 14.17	77.4–132.2	34.79 ± 5.7	25.2–48.1	28.59 ± 5.38	25.77–36.52	52.7 ± 10.4	42.3–97.5	17.4 ± 11.5	7.7–36.9

*BMIL* Body mass index loss, *EWL* Excess weight loss, *TBWL* total body weight loss

### Obesity Related Co-Morbidities

A significant improvement in obesity related co-morbidities was observed in the study group as shown in Table 3. The laboratory data during the study period is shown in Table 4.

**Table 3:** Remission rates of obesity associated medical problems after 5 years

Associated medical problems	No. before conversion	2 years after conversion (124)		No. at 5 years follow up	5 years after conversion (115)	
		**Number**	**Remission rate**		**Number**	**Remission rate**
Hypertension	34	12	64.7%	31	12	61.3%
Type 2 DM	26	4	84.6%	24	7	70.8%
Sleep apnea	68	23	66.2%	64	23	64.1%
Osteoarthritis	73	43	41.1%	68	43	36.8%
Dyslipidemia	37	9	75.7%	34	6	82.4%

*DM *Diabetes mellitus

**Table 4:** Important laboratory test data in the study group

Variable		Baseline	12 months	24 months	36 months	60 months
Fasting blood sugar(mg/dl)	Mean ± SD	176.67 ± 15.80	117.15 ± 35.50	103.42 ± 16.84	109.5 ± 7.2	105.4 ± 6.9
	Range	147–207	114–146	96–137	100–128	98–126
HbA1C (%)	Mean ± SD	8.88 ± 0.26	6.48 ± 0.45	5.76 ± 0.43	5.97 ± 0.40	5.91 ± 0.40
	Range	8.6–9.3	6.1–7.8	5.4–7.4	5.6–7.9	5.5–7.8
Hemoglobin (g/dl)	Mean ± SD	14.1 ± 2.3	12.4 ± 1.9	12.2 ± 1.8	12.5 ± 1.8	12.8 ± 0.5
	Range	12.8–15.1	11.6–13.8	11.3–13.5	11.6–13.7	10.2–14.1
Albumin (gm/dl)	Mean ± SD	4.4 ± 0.4	4.1 ± 0.5	3.85 ± 0.5	4.02 ± 0.5	4.05 ± 0.4
	Range	3.8–4.9	3.4–4.4	3.2–4.1	3.3–4.3	3.3–4.4
Cholesterol (mg/dL)	Mean ± SD	223.22 ± 34.63	182.58 ± 22.35	175.0 ± 20.5	178.0 ± 21.5	184.0 ± 21.5
	Range	139–288	163–228	155–215	160–220	164–262

### Primary Procedure-Related Results

We analyzed whether the type of primary operation influenced outcomes after cOAGB. We compared the operative and postoperative data between different primary procedure groups. The analysis revealed that the conversion rate to open surgery was not affected by the primary procedure type. In contrast, the operative times were significantly affected (longer in post-VBG patients) as shown in Table 5.

**Table 5:** Comparison between primary procedure groups regarding operative and post-operative data

Variable		LSG (26)		VBG (56)		AGB (31)		GCP (11)		*P*-value
		**No.**	**%**	**No.**	**%**	**No.**	**%**	**No.**	**%**	
Conversion		0	0.0	2	3.6	1	3.2	1	9.1	0.256^*^
Bleeding		1	3.8	0	0.0	0	0.0	0	0.0	0.316^*^
Leakage		1	3.8	1	1.9	0	0.0	1	9.1	0.359^*^
Wound complications		0	0.0	1	1.9	0	0.0	1	9.1	0.302^*^
Lung atelectasis/ chest infection		0	0.0	1	1.9	1	3.2	0	0.0	0.583^*^
Deep venous thrombosis		0	0.0	0	0.0	1	3.2	0	0.0	0.459^*^
Overall		2	7.6	3	5.7	2	6.4	2	18.2	0.0067^*^
Reoperation		0	0.0	1	1.8	0	0.0	1	9.1	< 0.0001^*^
Variable		**LSG**		**VBG**		**AGB**		**GCP**		**P-value**
Operation	**Mean ± SD**	98.60 ± 10.95		167.20 ± 33.18		114.60 ± 16.79		130.5 ± 29.46		< 0.0001^**^
time	**Range**	79–119		136–231		92–186		128–206		
Hospital stay	**Mean** **± SD**	4.60 ± 0.68		6.40 ± 2.46		3.80 ± 0.50		4.2 ± 1.68		< 0.0001^**^
	**Range**	4–16		4–21		3–4		4–17		

*Chi−Square test ** Student’s t−test

*LSG* Laparoscopic sleeve gastrectomy, *VBG* Vertical band gastroplasty, *GCP* Greater curvature plication, *AGB* Adjustable gastric band

The analysis also revealed significant differences between primary procedure groups in hospital stay (longer in the post-VBG group), reoperation rate, and overall complication rate (all higher in the post-GCP group), as shown in Table 5.

While the overall early complication rate showed a significant difference across the primary operation groups, analysis of individual complication types separately did not. This may be due to the small number of individual complications.

To identify the predictors of failure of cOAGB after restrictive BMS, the study group was divided into 2 subgroups [BMIL ≥ 20% (95) & BMIL ≤ 20% (20) after 5 years]. Univariate comparative analysis of all preoperative variables between both groups revealed that preoperative sex, type of primary procedure, type of failure, and presence of associated medical problems were significantly different between the groups, indicating that these variables are potential predictors of failure (Table 6).

**Table 6:** Univariate comparative analysis of all preoperative variables between failure and non-failure groups to detect the predictors of failure of cOAGB after restrictive bariatric surgeries

Variable		BMIL ≥ 20% (95)	BMI ≤ 20% (20)	*P* –value z
Sex	**Female (66)**	48 (50.5%)	18 (90%)	0.0003^*^
	**Male (49)**	47 (49.5%)	2 (10%)	
Type of primary operation	**LSG (23)**	17 (17.9%)	6 (30%)	0.0137^*^
	**VBG (52)**	46 (48.4%)	6 (30%)	
	**AGB (30)**	25 (26.3%)	5 (25%)	
	**GCP (10)**	7 (7.4%)	3 (15%)	
Mean pre conversion BMI (48.72 ± 13.47 kg/m^2^)		50.2 ± 12.37	44.6 ± 10.43	0.0618^* *^
Mean age (42.4 ± 15.81 years)		43.9 ± 7.58	40.6 ± 12.61	0.1230 ^* *^
Mean interval between primary operation and conversion (42.12 ± 15.81 months)		42.8 ± 11.17	41.5 ± 13.22	0.6479 ^* *^
Type of failure	**SIR (66)**	57 (60%)	9 (45%)	0.0341^*^
	**LPCD (49)**	38 (40%)	11 (55%)	
Associated medical problems	**HTN (31)**	19 (20%)	12 (60%)	0.0001 ^*^
	**DM (24)**	10 (10.5%)	14 (70%)	
	**Sleep apnea (64)**	49 (51.6%)	15 (75%)	
	**OA (68)**	54 (56.8%)	14 (70%)	
	**Back pain (83)**	71 (74.7%)	12 (60%)	
	**Dyslipidemia (34)**	21 (22.1%)	13 (65%)	

*Chi−Square test ** Student’s t−test

*HTN* Hypertension, *DM* Diabetes mellitus, *OA* Osteoarthritis BMI: Body mass index, *LSG* Laparoscopic sleeve gastrectomy, *VBG* Vertical band gastroplasty, *GCP* Greater curvature plication, *AGB* Adjustable gastric band, *SIR* Subclinical initial response, *LPCD* Late postoperative clinical deterioration

Then, significant variables were included in a multivariate stepwise logistic regression analysis to identify the independent predictors of failure. The logistic regression analysis revealed that the significant independent predictors of failure (achieving BMIL ≤ 20%) were (Fig. 3):

- Sex (female): strong positive effect (coef = 2.18, p < 0.001) – females were significantly more likely to achieve ≤ 20% BMIL.
- Presence of associated medical problems, especially:
  T2D: strong effect (coef = -2.10, p < 0.001).
  Dyslipidemia: (coef = -1.36, p < 0.001).
  Hypertension (HTN): (coef = -1.09, p = 0.005).
- Type of primary procedure: GCP and SG showed higher odds of failure compared to other procedures.
- LPCD as a failure type negatively influenced outcome (p = 0.001).

When interpreting the results of the multivariate analysis, we should take into account that there are a few failure events (20), which makes the results less reliable.

## Discussion

Restrictive BMS procedures remain the most commonly performed bariatric operations owing to their technical simplicity and low complication rate. The adverse effect of this trend is the rising revision rates, either due to SIR, LPCD, or postoperative complications [1].

There are no guidelines or consensus regarding the procedure of choice for conversion after restrictive BMS. cRYGB appears to be the procedure of choice for conversion because of its efficacy and safety profile; however, OAGB is gaining popularity as an alternative conversion procedure [10].

In our study, we aimed to assess the safety and effectiveness of OAGB as a conversion procedure for the management of SIR or LPCD after primary restrictive BMS procedures.

Our study affirms that cOAGB is technically feasible and reproducible. The mean operative time and intraoperative blood loss in our study were 155.3 min and 245 ml, respectively. Only 3.2% of operated patients were converted to open surgery, with no major intraoperative accidents. While some studies have shown operative times similar to ours (Almalki OM et al.) [2], others have reported significantly shorter times. In Parmar et al.’s systematic review of OAGB after failed restrictive operations, they reported a mean operative time of 119.3 min and a 1.2% conversion rate [11]. These results are better than ours, which may be explained by the higher proportion of VBG cases in our study (45.1% versus 9.7%). Our study showed that operative time was significantly longer in VBG cases due to the dense adhesions encountered.

Our early postoperative complications rate (7.6%) was better than rates reported in the literature. Almalki OM et al. reported a major complication rate of 11.1% and a minor complication rate of 6.2%. All their patients had either primary VBG or AGB [2]. In Petrucciani et al.‘s study, the overall postoperative complication rate was 13.5%, but all patients were post-VBG [12]. Studies comparing both cRYGB and cOAGB (Poublon et al. and Dayan et al.) reported lower complication rates after cOAGB [5, 13], and their cOAGB results were generally similar to ours.

After 5 years, 6.1% of our patients had biliary reflux, 7.8% had anemia, and 4.3% developed micronutrient deficiency. All these patients were managed conservatively with no need for reoperation. We had no cases of anastomotic stenosis/ulceration or internal hernia during the follow-up period. The rates of late post-cOAGB complications reported in the literature are variable. Some studies showed lower incidences of certain complications, such as the Musella et al. and Parmar et al. studies (0.3% and 1.9% anemia rates, respectively) [11, 14]. In contrast, others (Bruzzi et al.) reported an incidence of bile reflux similar to ours [10]. We did not have any cases of postoperative GERD in our study. The studies by Parmar et al. and Kermansaravi et al. showed post-cOAGB GERD rates of 3.29% and 7.4%, respectively. Both studies included patients with significant preoperative GERD, while we excluded them [11, 15].

The rates of late postoperative complications and nutritional outcomes seen in our study or reported in the literature are similar to those reported after cRYGB. In Hany et al.‘s study of both procedures after LSG, no significant difference was observed in nutritional outcomes or postoperative complications within 2 years of follow-up [16]. In their study, Pubeolon et al. had shown that intra-abdominal complications and internal herniation occurred less frequently after cOAGB. On the other hand, surgical intervention for biliary reflux was significantly higher in the cOAGB group. There was no significant difference between the two procedures in nutritional outcomes [5]. Our findings and the abovementioned literature support the impression that cOAGB is a safe procedure.

The EWL% in our study was 63.11% after 3 years and 52.7% after 5 years (BMIL% of 40.46% and 28.59%, respectively), with a 17.4% failure rate. This pattern of weight loss changes over the 5-year follow-up period (increase, then decrease) may be related to many factors, such as the patient’s feeding behavior, intestinal adaptation, and the loss of hormonal mechanisms induced by bypass surgery over the years [17–19].

Both Almalki OM et al. and Petrucciani et al. reported better results in their studies (EWL% after 5 years of 73% and 82.4%, respectively [2, 12]). In both studies, the primary operation was either VGB or AGB, which may explain their better results. In Parmar et al.‘s study, the EWL% was 71.6%, but they had a shorter follow-up (mean 20.8 months) and a slightly longer biliopancreatic limb (mean 226 cm) than ours [11].

Published literature comparing cRYGB and cOAGB has shown that weight-loss outcomes after cOAGB were comparable to or better than those after cRYGB. Pubeolon et al., Dayan et al., Dantas et al., and Rayman et al. studies reported significantly higher weight loss results after cOAGB [5, 13, 20, 21].

Multivariate logistic regression analysis done in our study revealed that female sex, associated medical problems, especially T2D, SG, and GCP (as primary procedures), and LPCD (as a failure type) were independent predictors of failure after cOAGB. In the study by Abu-Abeid et al., the independent predictors of optimal clinical response were male sex and higher preconversion BMI [2]. In Marwaha et al.‘s study, EWL > 40% after SG was an independent predictor of the below-average %EWL 1-year post-conversion, and BMI > 40 kg/m^2^ at the time of cRYGB was an independent predictor of both 1- and 2-year below-average %EWL post-conversion [22].

In our study, there was a significant improvement in obesity-related comorbidities after 5 years. Published literature confirms the potent metabolic effect of cOAGB. Parmar et al. reported a T2D remission rate of 80.5% and a hypertension remission rate of 63.7% with a median follow-up of 29.3 months [11]. Bruzzi M et al. reported a remission rate of 85% for T2D, 58% for hypertension, and 75% for dyslipidemia within 5 years of follow-up [10]. When comparing cOAGB with cRYGB, several studies (Pubeolon et al. and Hany et al. [5, 16]) found no significant difference between the two procedures in remission of obesity-associated medical problems.

Our study has several limitations. First, the absence of a direct control group makes it difficult to draw direct comparisons with other operations. Second, despite an overall sufficient number of patients, the wide heterogeneity of preconversion operations makes subgroup analysis less accurate. Third, the results of the multivariate logistic regression should be interpreted cautiously because the number of failure events was relatively low, which may have affected the accuracy of the results.

## Conclusion

OAGB is a safe and effective conversion procedure for the management of SIR and LPCD after restrictive procedures. It can be considered a good alternative conversion procedure after failed restrictive procedures.

## Data Availability

The data of this research are confidential.
